# Effects of *Lumbricus* sp. Extract Supplementation Methods and Levels in Fermented Feed on Digestibility, Growth, Gut Emptying, Blood Glucose and Phagocytic Parameters of Milkfish, *Chanos chanos* (Forsskal, 1775)

**DOI:** 10.21315/tlsr2026.37.3.11

**Published:** 2026-10-15

**Authors:** Siti Aslamyah, Yusdalifa Ekayanti Yunus, Nor Azman Kasan, Ayman Alameen Mohammed

**Affiliations:** 1 Aquaculture Study Program, Department of Fisheries, Faculty of Marine Sciences and Fisheries, Hasanuddin University, Jl. Perintis Kemerdekaan Km. 10, Tamalanrea, Makassar 90245, South Sulawesi, Indonesia; 2 Fish Hatchery Technology Study Program, Department of Aquaculture, Pangkep State Polytechnic of Agriculture, Makassar-Parepare Km. 83, Pangkep Regency, Indonesia; 3 Higher Institution Centre of Excellence (HICoE), Institute of Tropical Aquaculture and Fisheries (AKUATROP), Universiti Malaysia Terengganu, 21030 Kuala Nerus, Terengganu, Malaysia; 4 Department of Fisheries Science and Wildlife, College of Animal Production Science and Technology, Sudan University of Science and Technology, 6 Wehda street, District 7, Khartoum City, Sudan; 5 Research Center for Process Technology, National Research and Innovation Agency (BRIN), KST B.J. Habibie, Muncul Street, District Setu, 15314 South Tangerang, Banten, Indonesia

**Keywords:** *Chanos chanos*, Fermented Feed, Gut Emptying, *Lumbricus* sp. Extract, Supplementation Methods

## Abstract

This study evaluated the effects of different *Lumbricus* sp. extract supplementation methods and levels in fermented feed on the digestibility, growth, gut emptying, blood glucose dynamics and phagocytic parameters of milkfish juveniles. *Lumbricus* sp. extract was applied either by spraying onto dried pellets or by mixing into the feed before pelleting at 0, 100, 200 and 300 mL kg^−1^ feed. Experimental feeds were given to milkfish juveniles (17.80 ± 0.20 g) for 50 days in 1 m^3^ hapa cages in a pond with a water depth of approximately 70 cm at a daily feeding rate of 5% body weight. The 300 mL kg^−1^ treatment, in both supplementation methods, improved feed digestibility, specific growth rate and digestive enzyme activity (cellulase, α-amylase and protease) compared with lower levels. Phagocytic activity and leukocyte count also increased, while blood glucose peaked and declined more rapidly in fish receiving the highest extract level. The 300 mL kg^−1^ extract mixed into the feed before pelleting produced the fastest gut emptying, with complete emptying at the 5th postprandial hour. These findings indicate that *Lumbricus* sp. extract can be used as a functional additive in fermented milkfish feed, with 300 mL kg^−1^ as the most effective level under the present experimental conditions.

**HIGHLIGHTS**

- Supplementation with 300 mL kg^−1^ *Lumbricus* sp. extract improved feed digestibility, growth performance and digestive enzyme activity in milkfish juveniles.
- Mixing 300 mL kg^−1^ *Lumbricus* sp. extract into the feed before pelleting produced the fastest gut emptying, with complete evacuation at the 5th postprandial hour.
- *Lumbricus* sp. extract supplementation increased total leukocyte count and phagocytic activity, indicating enhanced innate immune responses in milkfish.

## INTRODUCTION

Milkfish (*Chanos chanos*) is an important aquaculture species contributing to aquatic food supply and livelihoods in the Western Pacific and Southeast Asia, where aquaculture continues to support food security and the availability of affordable animal protein (FAO 2024; Martinez *et al*. 2006). Advances in hatchery and nursery technologies, including controlled broodstock management, induced spawning, larval rearing, formulated starter feeds, live-feed management, aeration and improved water-quality monitoring, have supported the expansion of milkfish seed production in several Asian countries (Jaspe & Caipang 2011; Suparjo & Gozali 2020). However, in several least-developed and small-island countries, limited hatchery infrastructure, laboratory facilities, formulated feed access and technical capacity still constrain seed production and intensification, causing continued dependence on wild fry collection for pond restocking (Shadrack *et al*. 2021). These production constraints are closely linked to nutrition because reliable hatchery and grow-out performance depends not only on seed availability but also on nutritionally adequate and cost-efficient feeds that support growth, digestion and fish health.

In aquaculture, feed is one of the most important determinants of biological performance and production cost, particularly in intensive culture systems. Feed provides the energy and nutrients required for survival, growth and physiological function, but it can account for approximately 50% to 70% of production expenses (Yacout *et al*. 2016). Therefore, improving feed quality, digestibility and nutrient utilisation is essential for increasing the efficiency and sustainability of milkfish culture.

Feed quality can be improved through functional additives and fermentation-based processing. Feed additives such as enzymes, probiotics, prebiotics, vitamins, minerals, antioxidants and herbal ingredients may improve feed intake, feed utilisation, growth and immune function (Ahmad & Abdel-Tawwab 2011; Bagchi & Swaroop 2016). In tilapia, for example, green tea (*Camellia sinensis*) and *Silybum marianum* have been reported to enhance growth, resistance to *Aeromonas hydrophila*, antioxidant status and immune-related gene expression (Hassaan *et al*. 2019). Functional additives therefore provide a practical strategy to strengthen nutritional and health responses beyond conventional feed formulation (Alemayehu *et al*. 2018).

Earthworms, including *Lumbricus* sp., have attracted attention as both an alternative animal protein source and a functional feed additive because they contain bioactive metabolites and enzymes such as lumbrokinase, peroxidase, catalase, ligase and cellulase, as well as arachidonic fatty acids (Aslamyah *et al*. 2020; Bahadori *et al*. 2017; Istiqomah *et al*. 2017). In fish, *Lumbricus* sp. meal has been used as a partial fish-meal replacement in milkfish and tiger grouper (*Epinephelus fuscoguttatus*), supporting its potential role in aquafeed development (Aslamyah & Karim 2013; Widyasunu *et al*. 2013).

Non-conventional protein sources such as earthworms are increasingly relevant because aquaculture requires cost-effective ingredients with suitable nutritional value and reliable availability (Musyoka *et al*. 2019). Earthworm meal contains relatively high protein and a broad amino-acid profile, making it a potential substitute for fish meal in some aquafeed formulations (Janković *et al*. 2015; Jatmiko *et al*. 2018; Agustono & Anam Al-Arif 2019). However, the functional value of *Lumbricus* sp. extract may extend beyond protein contribution because its bioactive compounds may influence digestion, nutrient absorption and immune-related parameters.

Fermentation is another approach for improving feed efficiency because it can degrade complex feed components, reduce anti-nutritional factors and increase nutrient availability (Yang *et al*. 2021). For example, fermentation of sunflower meal with *Saccharomyces cerevisiae* and *Bacillus subtilis* increased crude protein, lipids and essential amino acids while reducing fibre, phytic acid, chlorogenic acid, caffeic acid and saponins (Hassaan *et al*. 2018). Similarly, fermentation of seaweed with *Bacillus* sp., *Rhizopus* sp. and *Saccharomyces* sp. improved dry matter digestibility, organic matter, protein content and nitrogen-free extract (Aslamyah *et al*. 2018). Combining fermentation with *Lumbricus* sp. extract may therefore improve feed digestibility, nutrient uptake and immunostimulatory responses in milkfish. Previous work reported improved growth performance when fermented milkfish feed was supplemented with *Lumbricus* sp. extract at 300 mL kg^−1^ feed, but the effects on digestive enzyme activity, gut emptying, postprandial blood glucose, leukocyte count and phagocytosis activity were not comprehensively evaluated (Aslamyah *et al*. 2019). Thus, the present study tested the hypothesis that supplementation method and level of *Lumbricus* sp. extract in fermented feed affect digestion-related and health-related responses in milkfish.

## MATERIALS AND METHODS

### Experimental Fish and *Lumbricus* sp. Extracts

This experiment was carried out using milkfish fingerlings (*C. chanos*) with an average size of 17.80 ± 0.20 g from Hasanuddin University Educational ponds in Barru Regency, South Sulawesi, Indonesia, while the *Lumbricus* sp. used in this research was obtained from local farmers in Makassar city, South Sulawesi, Indonesia.

The fish were stocked in 1 m^3^ hapa net cages set on the bottom of the pond in water with a depth of approximately 70 cm. The stocking density was 20 fish per hapa. Prior to trials, the experimental fish were acclimatised for one week to enable adaptation to the culture environment, and the base feed was provided at satiation for a week. At the end of the acclimatisation period, the fish were fasted for 24 h with the aim of eliminating any feed remaining in the digestive system. Over the experimental rearing period of 50 days, the fish in each unit were fed following the designated treatment feed formulation at 5% body weight per day with three daily feeding times: 07:00, 12:00 and 17:00 (Central Indonesian Time, GMT+8). Fish were weighed in grams by using the electronic top-loading balance every week to adjust for feed requirements and assess the growth performance of milkfish.

All procedures involving fish handling, sampling and euthanasia followed the ethical approval issued by the Research Ethics Committee, Universitas Muslim Indonesia, Makassar, Indonesia (Recommendation for Ethical Approval No. 404/A.1/KEP-UMI/III/2023). Fish selected for terminal sampling were handled gently using a scoop net, anaesthetised using an approved fish anaesthetic before dissection, and euthanised humanely before tissue collection to minimise stress and suffering.

The *Lumbricus* sp. extract was prepared following Hayati *et al*. (2011) with clarification of the cleaning and extraction steps. Live *Lumbricus* sp. were washed repeatedly with clean water to remove adhering soil and external debris. To remove remaining gut contents, the worms were z purged by holding them in cold clean water at 14°C for 24 h, after which the water was replaced until visible debris was no longer observed. Formic acid (80%) was then added at 3% of the wet weight of *Lumbricus* sp., and the mixture was homogenised using a blender. The homogenate was dried in an oven at 50°C for 12 h and sieved through a 40-mesh sieve (approximately 425 micrometers) to obtain a relatively uniform particle size. For extraction, one part *Lumbricus* sp. flour was mixed with 10 parts distilled water, homogenised, and heated in a water bath at 90°C for 30 min. The suspension was filtered, and the residue was re-extracted with distilled water at a ratio equivalent to 500 mL distilled water per 1 kg initial *Lumbricus* sp. material. The combined filtrates were homogenised, filtered again, and used as *Lumbricus* sp. extract according to the experimental treatment levels.

#### Experimental protocol

This study used a completely randomised factorial design with two factors. The first factor was administered: *Lumbricus* sp. extract, either sprayed on the artificial feed or mixed with the other feed ingredients before the feed was pelleted. The second factor was the rate of doses enriched with *Lumbricus* sp. extract (0 mL kg^−1^ as control, 100 mL kg^−1^, 200 mL kg^−1^ and 300 mL kg^−1^). All treatments were conducted in triplicate, for a total of 24 experimental units.

#### Experimental feed preparation

The experimental base feed was a formulated artificial feed prepared from locally available ingredients, including fish meal, soybean flour, copra meal, rice bran flour, Sargassum flour, cornflour, pollard, lipid sources and a vitamin-mineral mixture. The basal diet contained approximately 35.48% crude protein and 5.88% crude lipid after fermentation before *Lumbricus* sp. extract enrichment. The formulation of the experimental feed ingredients and the proximate composition of the diets before and after fermentation are presented in Tables 1 and 2, respectively. The trial feed production process began with drying the dry feed ingredients, which were then ground and weighed, except for lipids and the vitamin-mineral mixture.

The water quality in the culture pond was measured regularly throughout the experimental period. The parameters measured *in-situ* included temperature (thermometer TP3001), pH (pH meter KL-010), salinity (refractometer RHB-92ATC) and dissolved oxygen concentration (DO meter DO-5510HA). *Ex-situ* water quality parameters such as carbon dioxide concentration (titration) and ammonia (spectrophotometer, Genesys 150-Thermo Scientific) were measured in the Productivity and Water Quality Laboratory, Faculty of Marine Science and Fisheries, Hasanuddin University, Makassar, Indonesia.

The *in-situ* water quality parameters recorded in this study, including temperature, pH, salinity and dissolved oxygen, ranged from 26°C to 31°C, pH 6.8 to pH 7.1, 20 ppt to 25 ppt and 4.7 ppm to 5.6 ppm, respectively. For *ex-situ* parameters, carbon dioxide and ammonia ranged from 2.37 mg L^−1^ to 3.56 mg L^−1^ and 0.003 mg L^−1^ to 0.004 mg L^−1^, respectively.

### Assessment of Measured Parameters

#### Estimation of feed digestibility

Feed digestibility was measured using an indirect method, which involved mixing 0.6% chromium oxide (Cr_2_O_3_) evenly into the base feed. After 50 days, the experimental fish were transferred to a 45 L basin equipped with aeration. The experimental fish were fasted for 24 h, then fed with the experimental feed at the same feeding rate and frequency as stated previously. Faeces were collected daily using a faeces collector device for two weeks, starting two days after the first feed with the Cr_2_O_3_ laced feed. The collected faeces were dried in an oven at 60°C to avoid any changes in their compounds. The dried faeces were stored at −10°C for further analysis. Feed digestibility was calculated using the equation in Takeuchi (1988) as follows:


$$\text{Total digestibility}=100-(100\times \%\;{\text{Cr}}_{2}{\text{O}}_{3}\;\text{in feed}/\%\;{\text{Cr}}_{2}{\text{O}}_{3}\;\text{in faeces})$$

Where % Cr_2_O_3_ in feed is the percentage of Cr_2_O_3_ in the feed and % Cr_2_O_3_ in faeces is the percentage of Cr_2_O_3_ in the collected faeces.

#### Assessment of growth performance and digestive enzymes activity

In order to evaluate the growth performance of the milkfish, all fish from each treatment were measured once every week during the 50-day experiment. Feed intake (g fish^−1^) is the amount of feed given or supplied during the experimental period for each fish per gram and used for calculating the following equation:


$$\begin{array}{c} \text{Total weight gain (g}/\text{fish)}=\text{Final weight }(\text{g})-\text{Initial weight }(\text{g})\\ \text{Specific growth rate }(\%{\text{day}}^{-1})=\frac{\left(In\;final\;body\;weight-In\;initial\;body\;weight\right)}{\text{days}}\times 100\\ \text{Feed conversion ratio }(\text{FCR})=\frac{\text{Feed offered (g)}}{\text{Weight gain (g)}}\\ \text{Survival rate }(\%)=100\times \frac{\text{Final number of fish}}{\text{Initial number of fish}} \end{array}$$

At the end of the experiment, a total of five fish from each treatment were selected for analysis of digestive enzyme activity from their total intestinal contents. Preparing a crude enzyme extract was the first step in the analysis for cellulose, α-amylase and protease activity (Aslamyah 2006). The activity was conducted at a temperature of 0°C to 4°C with the aim of enzymes in an inactive condition. Digestive tract samples were washed with distilled water and dried with suction paper. Samples were taken as much as 1 g and crushed with a mortar until smooth and homogenised with 10 mL of cold distilled water, then centrifuged at 15,000 rpm for 20 min at 4°C, the supernatant was taken as a crude enzyme extract and used as a sample for measuring digestive enzyme activities.

Cellulase enzyme activity was determined using the 3,5-dinitrosalicylic acid (DNS) reducing-sugar method. A total of 80 microliters of crude enzyme extract and 720 microliters of 1% carboxymethyl cellulose substrate in citrate-phosphate buffer (pH 7.0) were incubated at 37°C for 60 min. The reaction mixture was then combined with 1,200 microliters of DNS reagent and heated in boiling water for 15 min. After cooling in an ice bath for 20 min, the reducing sugar formed was measured using a UV-Vis spectrophotometer at 540 nm. A heat-inactivated enzyme extract was used as the control. One unit of cellulase activity was defined as the amount of enzyme required to release 1 micromole of reducing sugar equivalent per minute under the assay conditions.

α-amylase activity was determined by mixing 1 mL of crude enzyme extract with 1 mL of 1% starch prepared in 0.05 M citrate buffer (pH 5.7). The mixture was incubated at 37 °C for 30 min, and the reaction was stopped by adding 2 mL of DNS reagent. The mixture was heated in boiling water for 5 min, cooled and centrifuged at 3,000 rpm for 5 min. The reducing sugar released was measured at 540 nm using a UV-visible spectrophotometer (Genesys 150, Thermo Scientific, China), following the reducing-sugar principle described by Miller (1959). One unit of α-amylase activity was defined as the amount of enzyme that produced 1 micromole of glucose equivalent per minute under the assay conditions.

Protease activity was measured based on Bergmeyer and Grassi (1983). Briefly, 1 mL of 2% casein solution was mixed with 1 mL of 0.01 M borate buffer (pH 8.0), 0.20 mL of 0.05 M hydrochloric acid and 0.20 mL of crude enzyme extract. The mixture was incubated at 37°C for 10 min, after which 2 mL of 0.1 M trichloroacetic acid was added to stop the reaction. After a further 10 min incubation, the mixture was centrifuged. A 1.5 mL aliquot of the filtrate was mixed with 5 mL of 0.4 M disodium carbonate and 1 mL of Folin-Ciocalteu reagent and then left to stand for 20 min. Absorbance was read at 578 nm. One unit of protease activity was defined as the amount of enzyme required to release 1 micromole of tyrosine equivalent per minute under the assay conditions.

#### Quantification of gastric emptying rate

Gut emptying rate was measured at the end of the study period over a 24 h postprandial period using a modified procedure based on Lee *et al*. (2000). Fish were fasted for 24 h and then fed to satiation with the assigned experimental diet. At each postprandial sampling time, fish were anaesthetised and euthanised humanely, and the digestive tract was dissected. The remaining feed in the gut was carefully removed and weighed. Three fish per treatment were sampled at each interval. Sampling was stopped when the gut contents of all sampled fish were empty. The percentage of feed remaining in the digestive tract was calculated as follows:


$$\text{Food remaining in the gut }(\%)=\frac{\text{Weight of feed remaining in the gut (g)}}{\text{Feed consumed (g)}}\times 100$$

#### Determination of blood glucose level

Blood samples were collected from the caudal vein using a 1 mL syringe and blood glucose levels were measured immediately using the strip method with a OneTouch Ultra Plus Flex SET glucometer. The unit was reported as mg dL^−1^.

#### Evaluation of blood and phagocytic parameters

Blood and phagocytic parameters were evaluated based on total leukocyte count and phagocytosis activity, both measured on days 25 and 50 of the experimental period. Total leukocytes were counted using a haemocytometer (WHI-30014 Assistant) under a microscope (Olympus BX51) following Blaxhall and Daisley (1973).


$$\text{Total leukocytes }(\text{cells }{\text{mm}}^{-3})=(\text{number of cells counted}\times \text{dilution factor}\times \text{chamber correction factor})$$

Phagocytosis activity was measured by mixing collected blood with 50 microliters of *Staphylococcus* sp. suspension (10^7^ cells mL^−1^). The mixture was incubated for 20 min, and 5 microliters of the suspension was placed on a clean glass slide using a micropipette, air-dried and fixed with methanol for 5 min to 10 min. The fixed blood smear on the glass slide was then immersed in Giemsa staining solution for 15 min to 20 min. The prepared slide was observed under a microscope (Olympus BX51). Phagocytosis activity was calculated following Rashid *et al*. (1997).


Phagocytosis activity (%)=[number of phagocytic cells/total cells counted]×100

### Statistical Analysis

The significant differences between treatments in feed digestibility, growth performance, digestive enzyme activity, leukocyte count and phagocytosis activity were tested using two-way analysis of variance (ANOVA) in SPSS version 21. Effects with a probability of *P* < 0.05 were considered significant. When significant differences were detected, Tukey’s honest significant difference test was used for multiple comparisons. Gut emptying rate and blood glucose levels were tabulated and analysed descriptively. Results are presented as mean ± standard deviation (SD).

## RESULTS

### Estimation of Feed Digestibility

The analysis of feed digestibility revealed significant differences (*P* < 0.05) among various treatments using different methods and rates of *Lumbricus* sp. extract enrichment as a milkfish feed additive. Results demonstrated that the highest feed digestibility was observed in milkfish feed enriched with 300 mL kg^−1^ *Lumbricus* sp. extract, regardless of the supplementation method employed. There was no significant difference in feed digestibility between supplementing with *Lumbricus* sp. extract at rates of 0 mL kg^−1^ (control), 100 mL kg^−1^ and 200 mL kg^−1^ for both enrichment methods (Fig. 1).

### Assessment of Growth Performance and Digestive Enzymes Activity

The results of initial fish weight, final weight, weight gain, SGR, feed intake, FCR and survival are presented in (Table 3). There were no differences in the initial weights of fish across all treatments (*P* > 0.05). After 50 days of feeding, there were significant differences (*P* < 0.05) in the growth parameters. Among the treated groups, fish fed with 300 mL kg^−1^ *Lumbricus* sp. extract enriched feed, whether mixed or sprayed, exhibited the highest final weights, weight gain, SGR and feed intake compared to other treatments. Fish fed 300 mL kg^−1^ *Lumbricus* sp. extract mixed into the feed exhibited the lowest FCR compared to other treatments. There were no significant differences in survival rate between all the treated groups (*P* > 0.05).

The activity of digestive enzymes, including cellulase, α-amylase and protease, is presented in Fig. 2. Cellulase activity in fish fed 200 mL kg^−1^ and 300 mL kg^−1^ *Lumbricus* sp. extract-enriched feed, whether mixed or sprayed, was higher than in the other treatments. Amylase and protease activities were also improved in fish fed 300 mL kg^−1^ *Lumbricus* sp. extract-enriched feed using both supplementation methods.

### Quantification of Gastric Emptying Rate

The result of gut emptying rate is presented in Table 4. The fastest emptying time was observed in the group receiving 300 mL kg^−1^ *Lumbricus* sp. extract mixed into the feed before pelleting, with complete emptying occurring by the 5th hour. In contrast, the other treatments, including 300 mL kg^−1^ *Lumbricus* sp. extract sprayed onto feed pellets and 0 mL kg^−1^, 100 mL kg^−1^ and 200 mL kg^−1^ in both methods, showed complete gut emptying after the 6th hour.

### Determination of Blood Glucose Level

The results of blood glucose levels over a 24 h period for *C. chanos* are presented in Figs. 3 and 4. Blood glucose levels were higher in fish fed 300 mL kg^−1^ *Lumbricus* sp. extract in both methods at 4 h postprandial compared with the other treatments. After 12 h, blood glucose levels decreased in almost all treatments and returned toward baseline levels.

### Evaluation of Blood and Phagocytic Parameters

Total leukocyte count and phagocytosis activity were assessed to evaluate the effects of fermented feed enriched with *Lumbricus* sp. extract on blood and phagocytic parameters in milkfish fingerlings. The results indicated significant differences among treatments (*P* < 0.05) for both total leukocyte count (Table 5) and phagocytosis activity (Table 6).

The total leukocyte count was measured on days 25 and 50. On day 25, fish fed 300 mL kg^−1^ *Lumbricus* sp. extract mixed into the feed showed the highest total leukocyte count followed by 300 mL kg^−1^ *Lumbricus* sp. extract sprayed onto the feed and the lowest value was observed in the control group. Similarly, on 50 days, fish fed 300 mL kg^−1^ *Lumbricus* sp. extract mixed into the feed showed the highest total leukocyte count, while those exposed to control diet exhibited the lowest levels among the other groups (*P* < 0.05).

Phagocytosis activity also exhibited significant differences among treatments. On day 25, *C. chanos* given feed enriched with *Lumbricus* sp. extract, whether sprayed onto feed pellets or mixed into the feed before pelleting, showed higher phagocytosis activity than the control treatment (*P* < 0.05). On day 50, fish fed 300 mL kg^−1^ *Lumbricus* sp. extract showed the highest phagocytic activity, followed by 200 mL kg^−1^ *Lumbricus* sp. extract in both methods. The lowest phagocytosis activity was observed in the control treatment and in fish given feed sprayed with *Lumbricus* sp. extract at 100 mL kg^−1^. These results indicate that both supplementation method and level influenced phagocytosis activity.

## DISCUSSION

This study aimed to investigate the effects of different methods and rates of enriching fermented feed with *Lumbricus* sp. extract on feed digestibility, growth performance, digestive enzyme activities, gastric emptying rate and blood glucose levels of milkfish (*C. chanos*) fingerlings.

The analysis of feed digestibility showed significant differences among the treatments, with the highest feed digestibility observed in milkfish feed enriched with 300 mL kg^−1^ *Lumbricus* sp. extract, regardless of the supplementation method employed. This suggests that the enrichment of milkfish feed with *Lumbricus* sp. extract can influence nutrient utilisation efficiency, and a dosage of 300 mL kg^−1^ appears to be ideal for increasing nutrient absorption. The study found that both spraying and mixing methods were equally effective in delivering the extract to the fish and facilitating nutrient absorption, as both methods resulted in consistent feed digestibility. The results of this study provide evidence that *Lumbricus* sp. extract can be an effective milkfish feed additive to improve nutrient utilisation efficiency. Studies have highlighted the capacity of microbes in the earthworm gut to respond to diverse saccharides, indicating the ongoing fermentation process in the earthworm gut (Meier *et al*. 2018). While the specific effects on feed digestibility may vary, these findings suggest that the use of *Lumbricus* sp. extract in fermented feed could potentially influence feed digestibility.

The evaluation of the growth parameters of milkfish in this study revealed the beneficial effects of *Lumbricus* sp. extract as a growth promotor. The growth performance findings were consistent with the feed digestibility findings, as fish fed 300 mL kg^−1^ *Lumbricus* sp. extract-enriched diet had the highest growth performance, indicating a direct link between improved feed digestibility and enhanced growth. One study found that supplementation of *Lumbricus* sp. extract in fermented feed had no significant effect on growth performance parameters, but the level of supplementation did have a significant effect on the survival and chemical composition of milkfish body (Aslamyah *et al*. 2019). Another study evaluated the quality of milkfish feed with different levels of fish meal substitution with earthworms (*Lumbricus* sp.) and found that the feed quality and nutrient content met the requirements for milkfish, suggesting that *Lumbricus* sp. can be substituted for fish meal in milkfish feed (Aslamyah & Karim 2012).

This study observed variations in the activity of digestive enzymes based on enrichment methods and rates. Higher enrichment rates were associated with increased enzyme activities, suggesting that *Lumbricus* sp. extract supplementation impacted digestive enzyme activity, leading to improved feed utilisation and growth. The positive correlation between enrichment rate and digestive enzymes activity in this study indicates enzymatic mechanisms underlying the enhanced feed utilisation and growth. Enzymes identified in the *Lumbricus* sp. extract, such as amylase, cellulase, lipase, endoglucanase and lumbrokinase, play crucial roles in promoting efficient carbohydrate digestion and utilisation, as well as repairing digestive tract tissue damage (Akazawa *et al*. 2016). This is in agreement with the study obtained by Haider *et al*. (2018) who observed that enzymes are vital catalysts in the digestion process and significantly influence the growth rate of fish. Their activity is profoundly influenced by the composition of the diet and the ingredients utilised in feed formulation.

The study showed that gut emptying was fastest when 300 mL kg^−1^ *Lumbricus* sp. extract was mixed into the feed before pelleting, resulting in complete emptying by the 5th hour. Because water temperature, fish size, feeding frequency and culture conditions were maintained similarly among treatments, these variables are unlikely to explain the observed differences. A more plausible explanation is that the two supplementation methods and extract levels may have affected the physical and chemical characteristics of the pellets, including nutrient availability, pellet surface coating, leaching, texture and enzyme accessibility. Mixing the extract before pelleting may have allowed a more uniform distribution of bioactive compounds within the feed matrix, thereby increasing contact between the extract and feed nutrients during digestion. This interpretation is consistent with the higher digestive enzyme activity and improved digestibility observed at the 300 mL kg^−1^ level. Protease, cellulase, and α-amylase activity may accelerate hydrolysis of protein, fiber-associated carbohydrates, and starch, respectively, facilitating nutrient release, absorption, and subsequent gut clearance. Therefore, the faster gut emptying in the mixed 300 mL kg^−1^ treatment appears to reflect diet processing and nutrient-digestive interactions rather than uncontrolled environmental or husbandry factors.

The observed variation in blood glucose levels of milkfish in response to *Lumbricus* sp. enrichment levels suggests a potential link between nutrient intake and stress. The post-feeding increase in glucose levels and subsequent decrease may reflect a complex interaction of multiple factors, including digestion and metabolism of the enriched feed, as well as the fish’s physiological stress response. Although elevated glucose levels may be indicative of stress, it is important to consider other factors, such as variations in nutrient uptake and utilization, that may be contributing to the observed patterns. The higher and earlier peak in glucose levels seen in the 300 mL kg^−1^ enrichment rate treatment may signify a more pronounced potential stress response, possibly due to higher nutrient content or specific characteristics of the enrichment method used. Further research is needed to investigate the underlying mechanisms and whether stressors play an important role in shaping glucose dynamics in milkfish. Blood glucose is the main source of energy and an important substrate for cell metabolism, especially in the fish brain (Malini *et al*. 2011). Differences in the composition of feed, especially fat and protein content, resulted in different blood glucose levels (Cheng *et al*. 2006), while gastric emptying rate and blood glucose levels both can be affected by insulin secretion (Heruc *et al*. 2018). *Lumbricus* sp. extract contains an insulin-like growth factor which is a mediator for the stimulation of several physiological processes, such as metabolism, growth and development (Aslamyah *et al*. 2020). This insulin-like hormone plays an important role when glucose enters the cell through the glycolysis process and the Krebs cycle; the resulting product becomes a source of energy (ATP).

Total leukocyte count and phagocytosis activity were assessed in this study to evaluate the effects of fermented feed enriched with *Lumbricus* sp. extract on blood and phagocytic parameters. These parameters were intentionally discussed together with growth, digestibility, gut emptying and blood glucose because improved nutrient utilisation may support both metabolic status and innate defence capacity. Fish fed *Lumbricus* sp. extract exhibited higher leukocyte counts and increased phagocytosis activity compared with the control group (*P* < 0.05), suggesting that the extract contributed to enhanced non-specific defense responses in milkfish.

## CONCLUSION

The findings indicate that Lumbricus sp. extract supplementation level had a stronger influence than supplementation method on most physiological responses in milkfish fingerlings. The highest level, 300 mL kg^−1^, improved feed digestibility, growth performance, digestive enzyme activity, gut emptying, postprandial blood glucose dynamics, leukocyte count and phagocytosis activity. Mixing the extract into the feed before pelleting produced the fastest gut emptying response. These findings support the use of *Lumbricus* sp. extract as a functional additive in fermented milkfish feed and suggest that 300 mL kg^−1^ is the most promising level under the present experimental conditions.

## Figures and Tables

**FIGURE 1:**
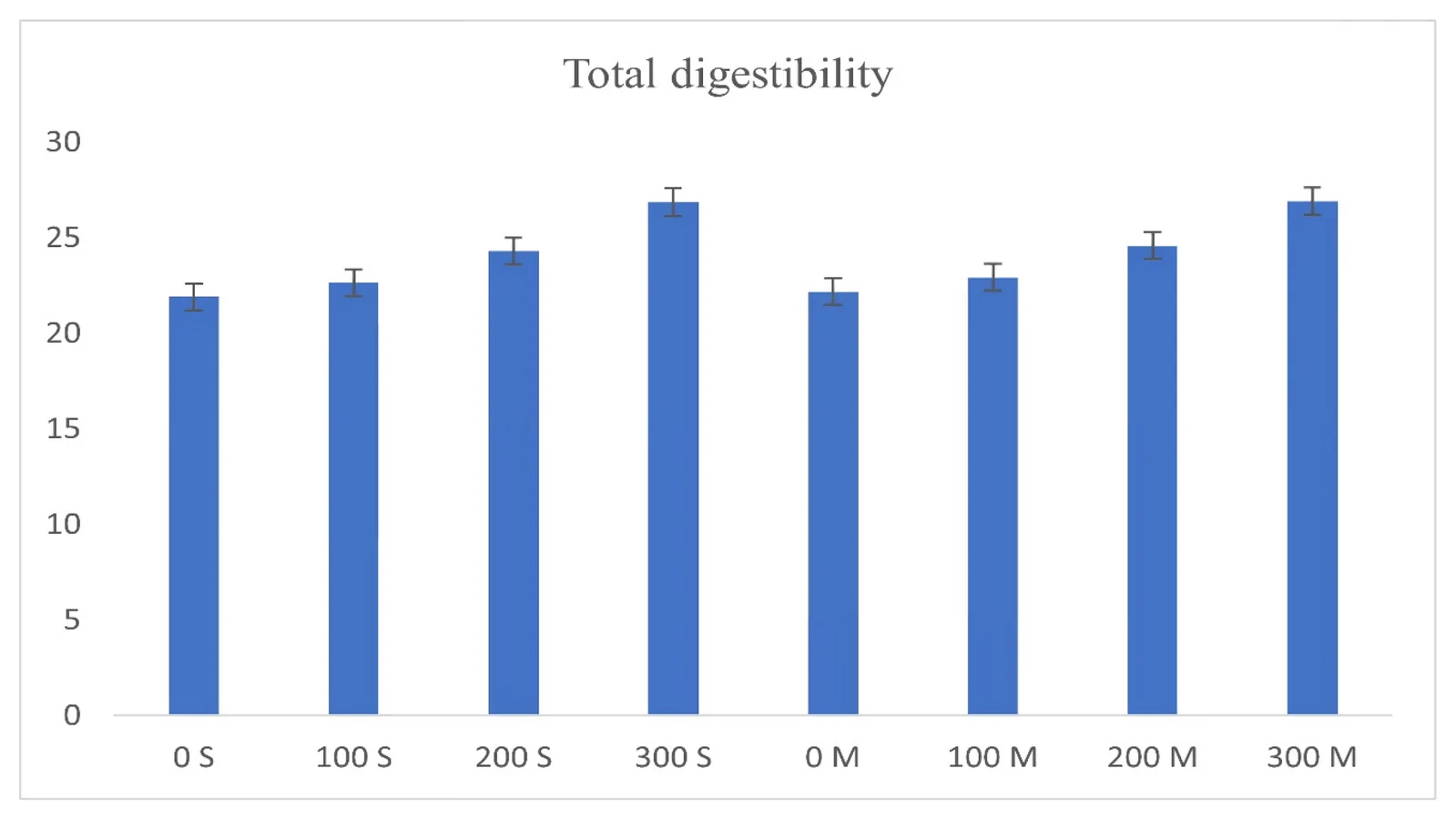
Total digestibility (%) of experimental fish. *Notes*: S = Sprayed onto the feed pellets; M = Mixed into the feed before pelleting

**FIGURE 2:**
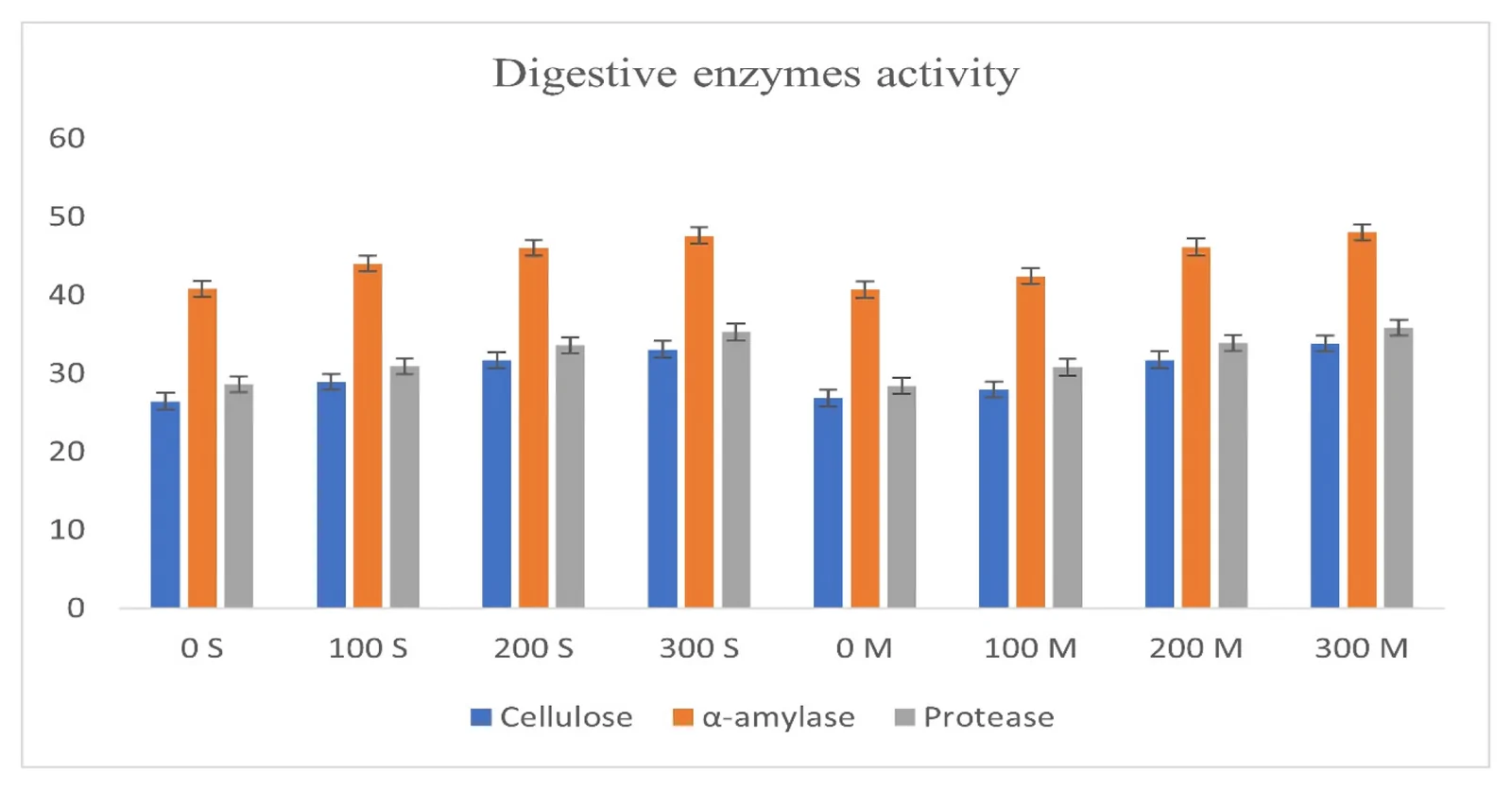
Digestive enzymes activity (IU) milkfish fingerlings given feed enriched with *Lumbricus sp*. extract using different methods and enrichment rates. *Notes*: S = Sprayed onto the feed pellets; M = Mixed into the feed before pelleting

**FIGURE 3:**
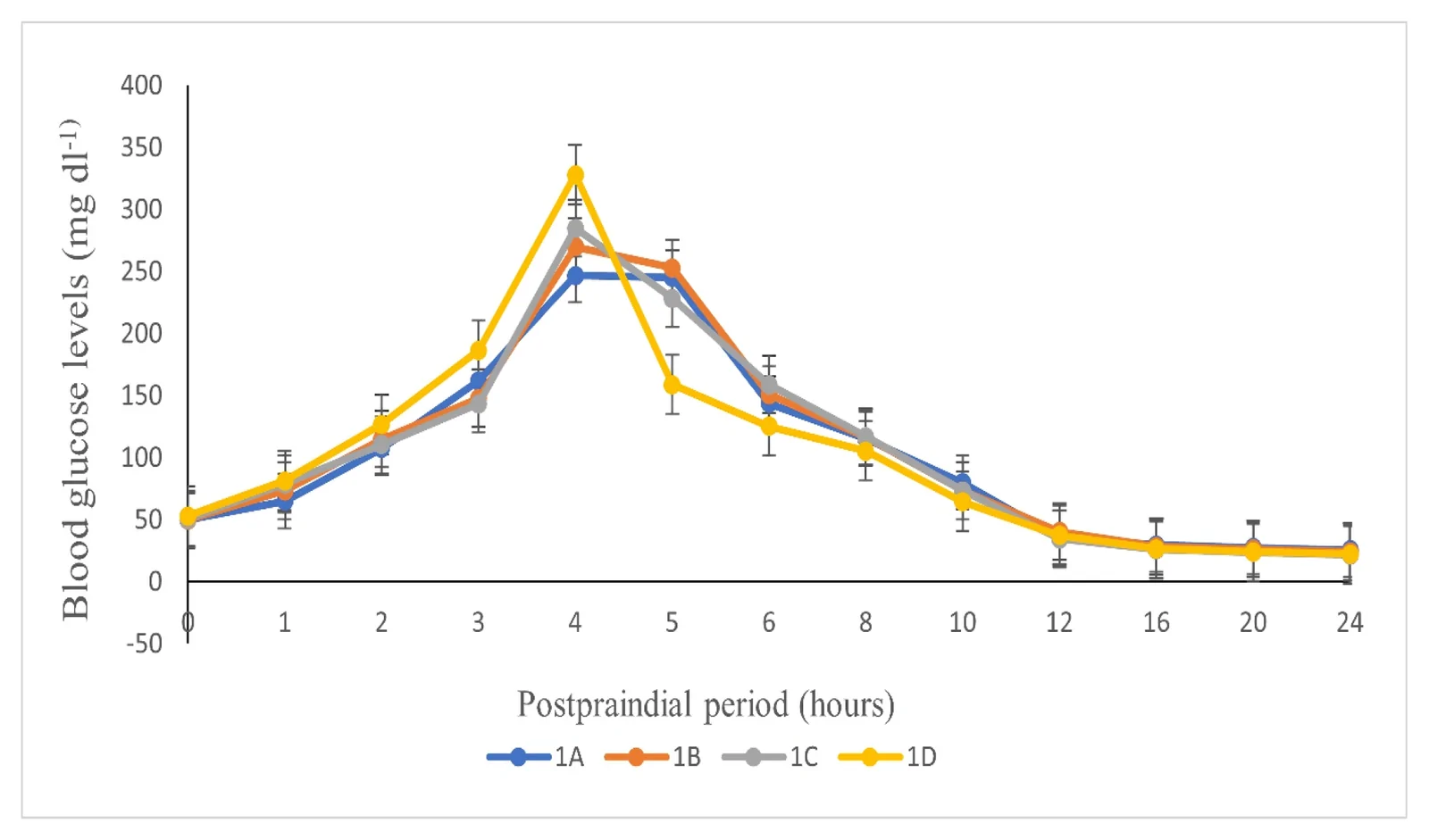
Mean blood glucose levels (mg/dL^−1^) by postprandial sampling period (hours) of milkfish fingerlings given feed enriched with *Lumbricus sp*. extract using different methods and enrichment rates: A. Sprayed onto the pelleted feed (1A = 0 mL kg^−1^ (control), 1B = 100 mL kg^−1^, 1C = 200 mL kg^−1^, 1D = 300 mL kg^−1^).

**FIGURE 4:**
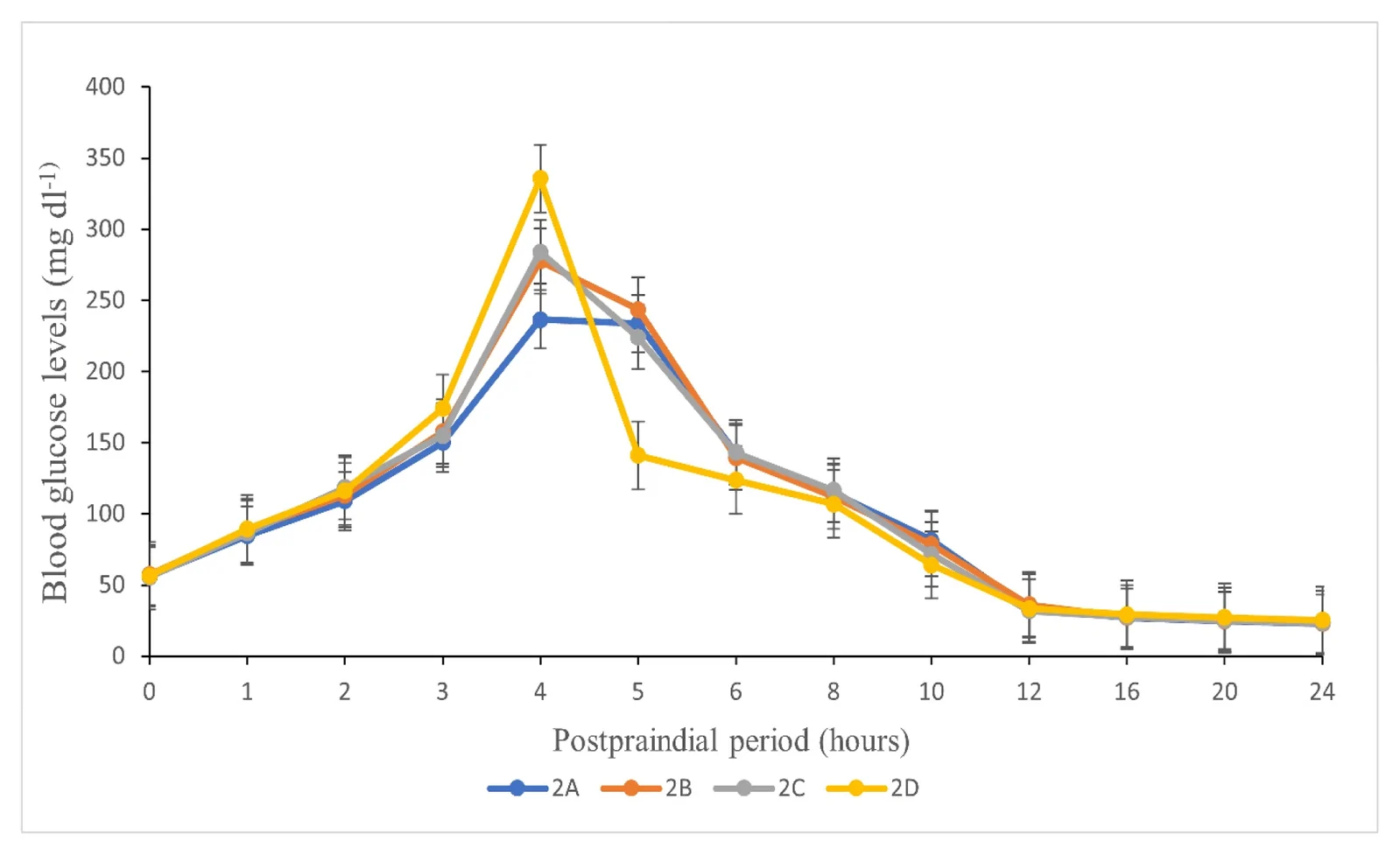
Mean blood glucose levels (mg/dL^−1^) by postprandial sampling period (hours) of milkfish fingerlings given feed enriched with *Lumbricus sp*. extract using different methods and enrichment rates: B. Mixed into the feed before pelleting feed (2A = 0 mL kg^−1^ (control), 2B = 100 mL kg^−1^, 2C = 200 mL kg^−1^, 2D = 300 mL kg^−1^).

**TABLE 1:** Formulation of the experimental feed ingredients.

Feed ingredients*	Protein levels (%)	Weight (%)
Fish meal	53.2	30
Soybean flour	37.5	14
Copra meal	18.3	10
Rice bran flour	10.3	10
*Sargassum* flour	5.4	10
Cornflour	9.5	10
Pollard	11.9	10
Lipid**		3
Vitamin and mineral mix***		3
Total		100

*Notes*

* All feed ingredients were obtained from local markets at Makassar city, South Sulawesi, Indonesia;

** Fish oil and corn oil in a 2:1 ratio;

*** Vitamin and mineral mix composition per 10 kg: Vitamin A 12,000,000 IU; Vitamin D 2,000,000 IU; Vitamin E 8,000 IU; Vitamin K 2,000 mg; Vitamin B_1_ 2,000 mg; Vitamin B_2_ 5,000; Vitamin B_6_ 500 mg; Vitamin B_12_ 12,000 μg; Ascorbic acid 25,000 mg; Calcium-D-Pantothenate 6,000 mg; Niacin (vitamin B_3_) 40,000 mg; Choline Chloride 10,000 mg; Metheonine 30,000 mg; Lysine 30,000 mg; Manganese 120,000 mg; Iron 20,000 mg; Iodine 200 mg; Zinc 100,000 mg; Cobalt 200,000 mg; Copper 4,000 mg; Santoquin (antioxidant) 10,000 mg; Zinc bacitracin 21,000 mg.

**TABLE 2:** Results of proximate analysis of experimental feeds

Proximate analysis	Composition (% dry weight)								

	Before fermentation	After fermentation							

		Sprayed onto the feed pellets (mL kg^−1^)				Mixed into the feed before pelleting (mL kg^−1^)			

		0	100	200	300	0	100	200	300
Moisture content	11.54	11.02	11.42	11.82	12.22	11.02	11.27	11.52	11.77
Crude protein	26.43	35.48	35.69	35.89	36.09	35.48	3571	35.94	36.16
Crude lipid	8.37	5.88	5.90	5.92	5.93	5.88	5.90	5.92	5.94
Ash content	24.22	14.22	14.17	14.12	14.07	14.22	14.16	14.10	14.05
Crude fibre	7.45	5.34	5.31	5.28	5.26	5.34	5.31	5.28	5.25
Nitrogen-free extract (NFE)	33.53	39.08	38.94	38.79	38.66	39.08	38.92	38.76	38.61
Digestible energy/DE (kcal kg^−1^) *	2,627.52	2,807.87	2,813.16	2,818.37	2,823.49	2,807.87	2,813.78	2.819.58	2,825.28
C/P (Calories DE g^−1^ protein)	9.94	7.99	7.96	7.93	7.90	7.99	7.96	7.92	7.89

*Notes*:

* Calculated based on the energy equation (NRC 1988): 1 g carbohydrate = 2.5 kcal DE; 1 g protein = 3.5 kcal DE; 1 g lipid = 8.1 kcal DE.

**TABLE 3:** Initial fish weight, final fish weight, weight gain, SGR, feed intake, FCR and survival % of milkfish fingerlings fed on feed enriched with *Lumbricus* sp. extract.

Parameter	Enrichment method							

	Sprayed onto the feed pellets (mL kg-1)				Mixed into the feed before pelleting (mL kg-1)			

	0	100	200	300	0	100	200	300
IW (g)	17.04 ± 0.05^a^	17. 03 ± 0.04^a^	17.01 ± 0.05^a^	17.02 ± 0.06^a^	17.01 ± 0.07^a^	17.00 ± 0.02^a^	17.00 ± 0.06^a^	17.01 ± 0.06^a^
FW (g)	25.85 ± 3.09^a^	25.85 ± 3.09^a^	27.01 ± 1.75^b^	30.30 ± 2.54^c^	26.38 ± 1.44^a^	25.30 ± 1.48^a^	27.37 ± 1.05^b^	33.94 ± 4.00^c^
WG (g)	8.82 ± 3.06^a^	8.83 ± 3.06^a^	10.01 ± 1.78^b^	13.28 ± 2.46^b^	9.38 ± 1.50^a^	8.30 ± 1.48^a^	10.37 ± 1.08^b^	16.93 ± 4.03^c^
SGR (% day^−1^)	0.83 ± 0.23^a^	0.83 ± 0.23^a^	0.92 ± 0.13^a^	1.15 ± 0.16^b^	0.88 ± 0.12^a^	0.79 ± 0.12^a^	0.95 ± 0.08^a^	1.37 ± 0.25^b^
FI (g)	601.08 ± 232.21^a^	796.28 ± 64.75^a^	581.03 ± 63.13^a^	584.65 ± 61.55^a^	719.31 ± 95.98^a^	629.57 ± 210.82^a^	609.68 ± 44.48^a^	692.53 ± 107.09^a^
FCR	4.31 ± 1.13^a^	4.96 ± 1.32^a^	3.83 ± 0.73^a^	3.05 ± 0.64^a^	4.39 ± 0.59^a^	4.85 ± 1.09^a^	3.74 ± 0.28^a^	2.58 ± 0.76^b^
SR %	68.33 ± 29.30^a^	93.33± 7.63^a^	66.67± 7.64^a^	66.67± 7.64^a^	83.33 ± 12.58^a^	71.67 ± 27.53^a^	70.00 ± 5.00^a^	80.00 ± 13.22^a^

*Notes:* Different superscript letters indicate significant differences between treatments at the 95% confidence level (*P* < 0.05). IW = Initial weight, FW = Final weight, WG = Weight gain, SGR = Specific growth rate, FI = Feed Intake, FCR = Feed conversion ratio, SR = Survival rate.

**TABLE 4:** Gastric emptying rate (%) of milkfish fingerlings given feed enriched with *Lumbricus* sp. extract.

Enrichment method	Enrichment rate (mL kg^−1^)	Gastric emptying rate (%)					

		Postprandial period (hours)					

		1	2	3	4	5	6
Sprayed onto the feed pellets	0	89.09 ± 3.78	76.51 ± 1.76	52.41 ± 6.05	32.53 ± 2.3	11.45 ± 0.46	0
	100	91.41 ± 5.28	76.69 ± 1.94	52.76 ± 3.93	32.52 ± 0.59	11.04 ± 1.83	0
	200	89.29 ± 1.86	73.81 ± 1.24	52.98 ± 5.0	31.55 ± 1.37	10.71 ± 0.18	0
	300	87.35 ± 2.77	70.48 ± 2.05	51.2 ± 3.39	31.33 ± 5.65	4.82 ± 6.73	0
Mixed into the feed before pelleting	0	92.41 ± 1.93	70.25 ± 6.8	56.96 ± 6.14	32.91 ± 2.38	12.03 ± 1.11	0
	100	88.75 ± 0.4	78.75 ± 2.52	55.63 ± 2.85	32.5 ± 2.92	11.88 ± 1.3	0
	200	86.5 ± 3.12	71.17 ± 14.0	47.24 ± 10.08	30.06 ± 7.03	9.20 ± 4.10	0
	300	86.96 ± 0.76	77.02 ± 0.68	42.92 ± 13.55	30.47 ± 8.17	0	0

**TABLE 5:** Mean total leukocyte counts of milkfish fingerlings given fermented feed enriched with *Lumbricuss* sp. extract.

Enrichment method	Enrichment rate (mL kg^−1^)	Total leukocyte count (cells mm^−3^)	

		Day 25 (mean ± SD)	Day 50 (mean ± SD)
Sprayed onto the feed pellets	0	287.50 ±.00^a^	950.00 ± 38.01^a^
	100	327.08 ±14.43^b^	1254.17 ± 41.61^c^
	200	420.83 ± 26.02^c^	1393.75 ± 76.80^e^
	300	1355.55 ± 278.11^f^	1475.00 ± 43.30^g^
Mixed into the feed before pelleting	0	229.17 ± 31.46^a^	1106.25 ± 86.83^b^
	100	343.75 ± 39.03^b^	1293.75 ± 62.50^d^
	200	698.61 ± 182.70^d^	1433.33 ± 36.08^f^
	300	1956.25 ± 43.75^e^	1533.33 ± 47.32^h^

*Note*: different superscript letters indicate significant between treatment differences in total leukocyte count at the 95% confidence level (P < 0.05).

**TABLE 6:** Mean phagocytosis activity of milkfish fingerlings given fermented feed enriched with *Lumbricus* sp. extract.

Enrichment method	Enrichment rate (mL kg^−1^)	Phagocytosis activity (%)	

		Day 25 (mean ± SD)	Day 50 (mean ± SD)
Sprayed onto the feed pellets	0	20.67 ± 6.35^a^	56.67 ± 1.15^c^
	100	56.33 ± 3.79^c^	58.33 ± 11.59^c^
	200	52.00 ±.00^c^	79.00 ±.00^d^
	300	52.00 ± 8.66^c^	80.00 ±.00^d^
Mixed into the feed before pelleting	0	18.33 ± 10.21^a^	36.67 ± 2.08^a^
	100	42.00 ±.00^b^	48.00 ±.00^b^
	200	46.67 ± 5.11^b^	54.00 ±.00^b^
	300	52.33 ± 0.58^c^	73.33 ± 5.51^d^

*Note*: Different superscript letters indicate significant between treatment differences in total leukocyte count at the 95% confidence level (*P* < 0.05).
